# Simultaneous Application of Raman and Laser-Induced Breakdown Spectroscopy in the Gas Phase with a Single Laser and Detector

**DOI:** 10.1177/00037028241227459

**Published:** 2024-01-31

**Authors:** Johannes Kiefer

**Affiliations:** 1 University of Bremen, Technische Thermodynamik, Bremen, Germany; 2 University of Bremen, MAPEX Center for Material and Processes, Bremen, Germany

**Keywords:** Raman, laser-induced breakdown spectroscopy, LIBS, pulse stretcher, gas analysis, composition

## Abstract

Raman spectroscopy and laser-induced breakdown spectroscopy (LIBS) are powerful tools for molecular and elemental analysis, respectively. Their combined application, however, is challenging due to the differences in the signal generation and detection characteristics. This note proposes three experimental schemes for the simultaneous application of Raman and LIBS for gas-phase diagnostics. Ring-cavity optical pulse stretchers facilitate shaping suitable pulse pairs from a Q-switched laser that enables the quasi-simultaneous detection of the Raman and LIBS signals on a single detector.

## Graphical abstract

**Graphical abstract:**
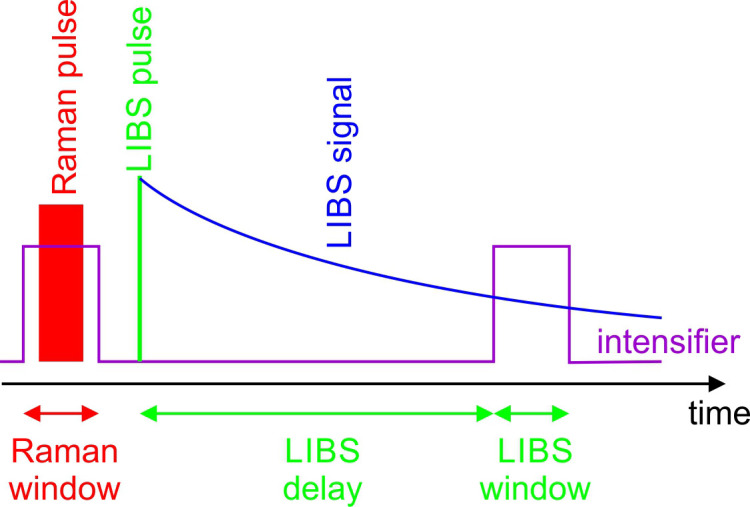
This is a visual representation of the abstract.

## Introduction

The remote elemental and molecular chemical analysis of gases, liquids, and solids is a common task in many areas of natural sciences and engineering. However, it is difficult to find a single method that can provide both pieces of information. Consequently, the combination of two analytical techniques seems to be the most promising approach.

In order to obtain a comprehensive picture of the chemistry of a sample, methods that in principle allow sensing all target species at once are desirable. Regarding the elemental analysis, laser-induced breakdown spectroscopy (LIBS) is an excellent candidate.^1,2^ In a LIBS experiment, a high-intensity laser pulse is used to create a local optical breakdown of the sample. The molecules and atoms present are atomized and ionized via multiphoton absorption and cascade avalanche ionization. A plasma is formed that emits optical signatures according to its elemental composition. At the molecular analysis end, Raman scattering is a versatile method.^3,4^ The sample is irradiated by a laser and the inelastically scattered light represents the signal, which constitutes the vibrational and rotational fingerprints of the molecules in the sample. In both methods, LIBS and Raman, the signal generation does not require the excitation with light of certain specific wavelengths. In turn, signals from virtually all species in the sample are produced at the same time. Hence, combining LIBS and Raman spectroscopy is a very promising approach to obtain a full chemical picture of a sample. Therefore, it is not surprising that these two tools have already seen quite a few joint applications.^5–8^ The most prominent example is certainly the Mars *Perseverance* rover that carries Raman and LIBS instruments for analyzing Martian soil.^9^

In those joint applications, the Raman and LIBS are often separated from each other: there are two different laser sources and two detection systems. This is not a problem, when samples such as minerals are analyzed as the measurements can be carried out subsequently to avoid any cross interferences; non-destructive Raman comes first, of course. When the sample takes part in a transient process, however, combining Raman and LIBS is particularly challenging as a short measurement time is required. A single-shot Raman/LIBS approach can still only produce reasonable results when the Raman measurement is performed before the LIBS breakdown takes place. Furthermore, the requirements for the light source are quite different for the two methods: LIBS needs a short pulse with high intensity in order to exceed the breakdown threshold of the sample while Raman benefits from pulses with high energy but with an intensity below the breakdown threshold. In order to achieve the latter, optical pulse stretchers are often used to enable Raman measurements with Q-switched lasers. In such a pulse stretcher, the initial high-intensity pulse with a few nanoseconds duration is shaped into a temporally stretched pulse.^10,11^

In this note, we propose a method that enables the combination of Raman and LIBS in the gas phase utilizing a Q-switched neodymium-doped yttrium aluminum garnet (Nd:YAG) laser, an optical ring-cavity pulse stretcher, and an optical delay line. Two different pulse stretchers, a conventional one and a polarization-controlled alternative, are considered.

## Combined Raman/LIBS Approach

**Figure 1.**
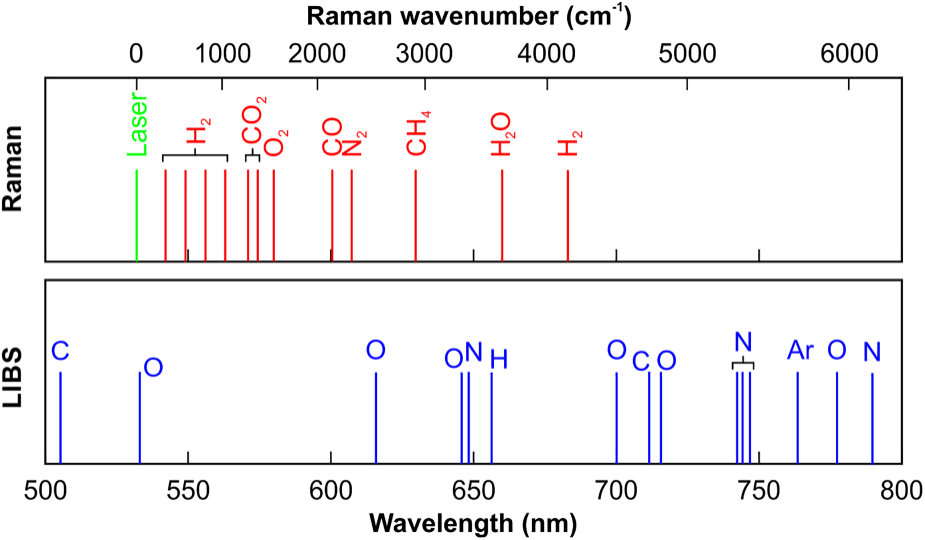
Schematic Raman and LIBS spectrum of typical gases (e.g., in air and fuels). Note that the Raman wavenumber scale is nonlinear as it corresponds to the linear wavelength scale.

In order to perform combined Raman and LIBS measurements with a single Nd:YAG laser, the different wavelength options need to be considered. While the spectral position of the LIBS signal will appear independent of the laser wavelength, the Raman spectrum is directly linked with the incident light. The lower panel of Figure 1 illustrates a schematic gas-phase LIBS spectrum. As all relevant elements (here C, O, N, H, and Ar) produce signatures between 500 and 800 nm, this is the spectral range that needs to be detected. At the same time, this suggests the use of the second harmonic of the Nd:YAG laser at 532 nm as the Raman laser as the signals will appear between 540 and 700 nm. Consequently, both spectra can in principle be detected with a single spectrograph and detector. This leaves two options for LIBS: 532 and 1064 nm. Both will be considered below.

**Figure 2.**
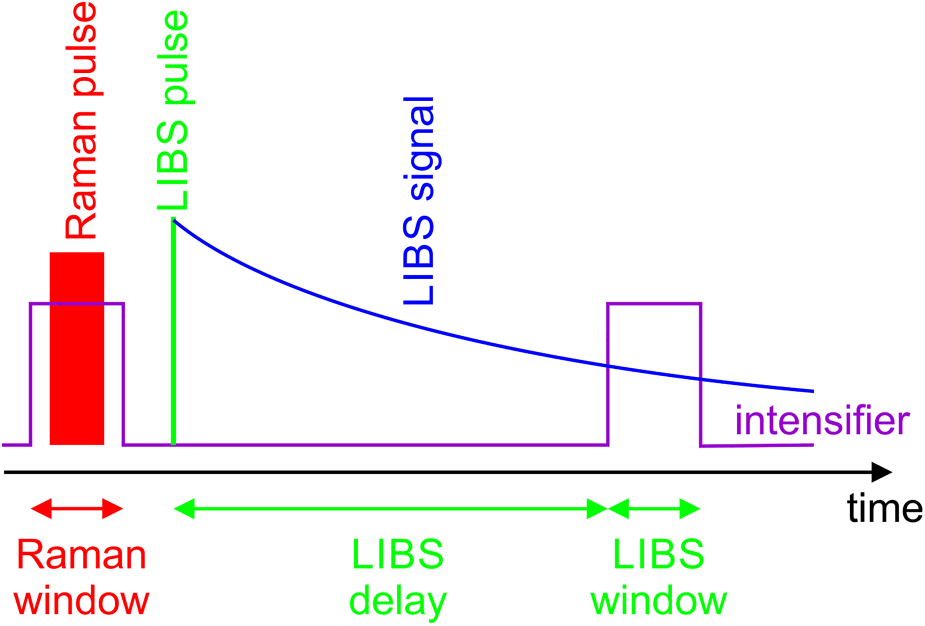
Time scheme of combined Raman/LIBS measurement.

The temporal sequence of the combined Raman/LIBS measurement is the second important aspect. As discussed above, the molecules are destroyed during the LIBS breakdown. Therefore, the Raman measurement must take place before the LIBS laser pulse arrives. Also, the difference in signal levels may be an issue as the LIBS signal is typically strong enough to be seen with the naked eye, while the Raman signal is inherently weak. So recording both signals with one detector is challenging. However, we can take advantage of the temporal behavior of the LIBS signal, which decreases exponentially with time after the laser pulse. To take those points into account, the time scheme illustrated in Figure 2 is proposed. A first prolonged Raman pulse reaches the probe volume to generate the Raman signal, which is recorded by an intensified camera. The intensifier is then switched off for the LIBS pulse to arrive and the period during which the unspecific plasma continuum emission from bremsstrahlung is released. It is then switched on again to record species-specific LIBS signals in a time window that ensures the signal amplitudes do not significantly exceed the Raman signal.

From the above considerations, it becomes clear that the combined Raman/LIBS measurement requires a pair of laser pulses, the first one with high energy and low intensity for the Raman part and the second one with high intensity for LIBS. Using a standard Q-switched Nd:YAG laser means that the Raman pulse needs to be 532 nm and the LIBS pulse 532 or 1064 nm radiation. In order to reduce the peak intensity of typical Q-switched lasers for Raman applications optical pulse stretchers are commonly used. There are two main concepts for such pulse stretchers, a ring cavity with a beamsplitter^11^ or polarization control, polarization-controlled optical ring cavity (PORC).^10^ Both are capable of producing the lengthened Raman pulse. The LIBS pulse can just be a part of the original laser pulse. However, simply using the pulse stretcher is not enough as the Raman pulse needs to arrive sufficiently early before the LIBS pulse. This calls for installing an optical delay line.

**Figure 3.**
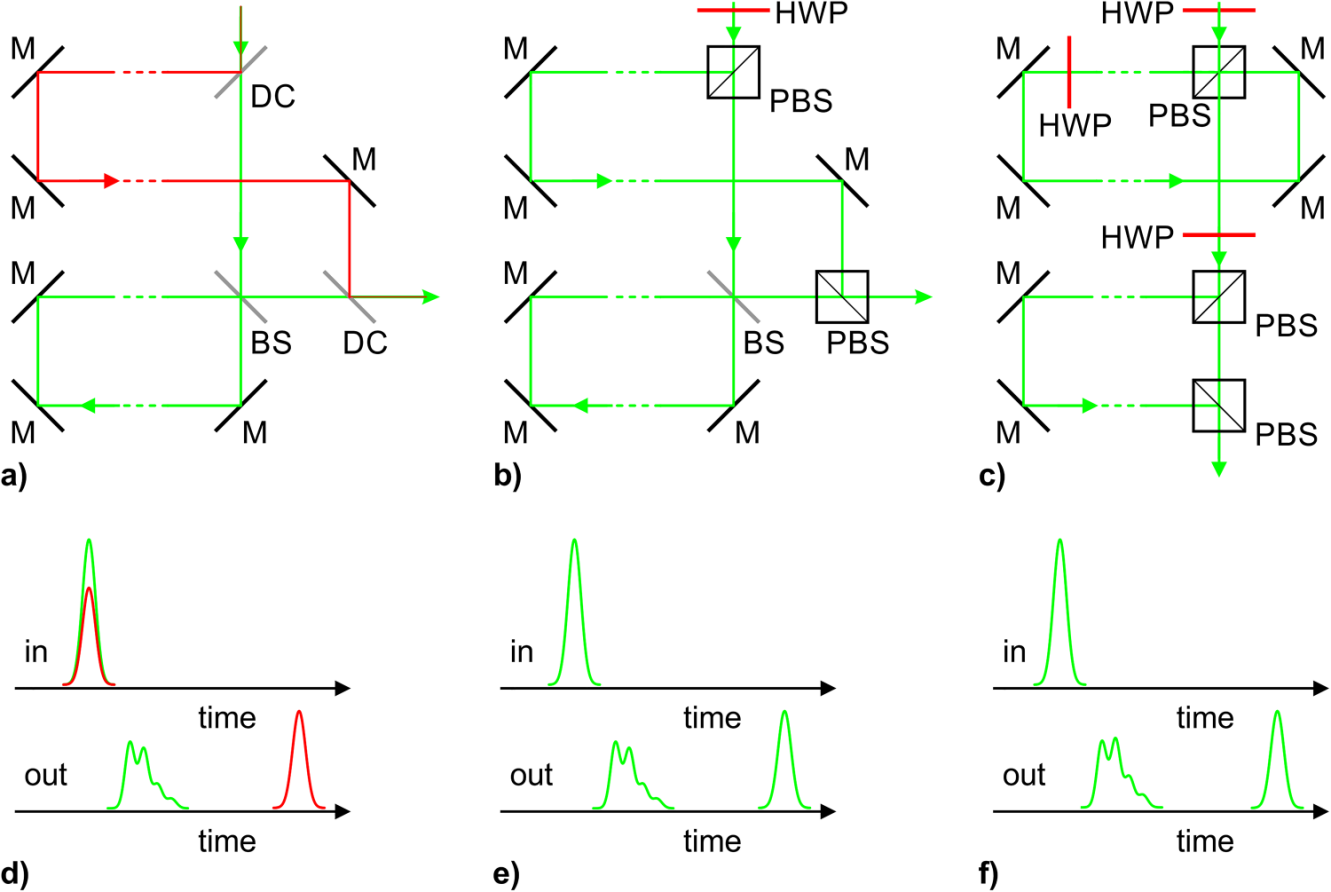
Schematic arrangements of the optical delay line and pulse stretcher, (a–c), and the incoming and outgoing pulse sequences, (d–f); the red and green beams in panel (a) indicate the 1064 and 532 nm wavelengths, respectively. M: mirror; DC: dichroic mirror; BS: beamsplitter; HWP: half-wave plate; PBS: polarizing BS.

Three suitable arrangements are illustrated in Figures 3a–3c. Figures 3d–3f show the incoming and outgoing pulse sequences. Figure 3a presents the variant that utilizes the first harmonic residual of the Nd:YAG laser at 1064 nm for LIBS, while Figures 3b and 3c show the single-wavelength options with the two different pulse stretcher concepts. The initial half-wave plates are used to control the energy split between the Raman and LIBS pulses. A theoretical optimization showed that the PORC concept in Figure 3c allows the Raman pulse to contain ∼7% higher energy, which eventually means 7% more Raman signal.

## Proof-of-Concept Experiment

A simple proof-of-concept experiment was carried out in room air using the PORC pulse stretcher concept in Figure 3c. A Nd:YAG laser (Quantel, Q-Smart 850; 532 nm, 6 ns) was employed as the light source and an intensified charge-coupled device (CCD) camera (Andor, iStar) was the detector, which was attached to an imaging spectrograph (Princeton Instruments, Acton). The delay line was about 20 m long to provide sufficient temporal separation between the pulses (∼60 ns). The laser was operated at a 5 Hz repetition rate. The intensifier of the camera could be controlled with a 500 kHz repetition rate, so it was switched on for 100 ns during the Raman pulse and then again after 2 µs for another 100 ns for the LIBS detection. The electronics of the CCD chip are significantly slower than the intensifier, so the CCD chip accumulated signal from both detection windows to yield a single combined Raman/LIBS spectrum. A schematic drawing of the setup and further details are provided in the Supplemental Material (see Figure S1).

**Figure 4.**
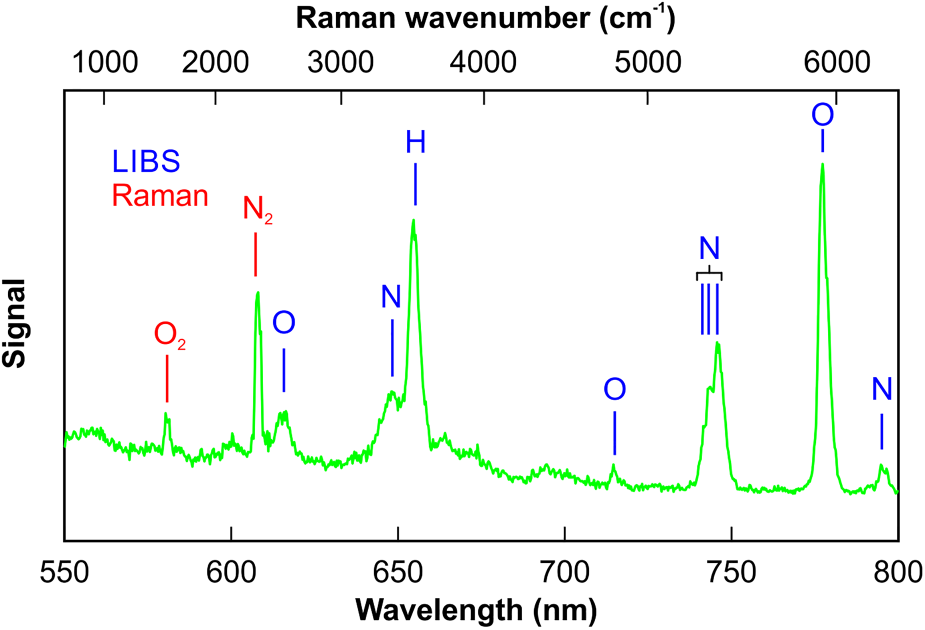
Experimental Raman/LIBS spectrum recorded in room air.

The room air Raman/LIBS spectrum obtained is shown in Figure 4. It shows the Raman signatures of molecular oxygen and nitrogen together with the LIBS lines of atomic/ionic nitrogen, oxygen, and hydrogen. The hydrogen peak results from the presence of ambient moisture (ambient conditions were 27% relative humidity and 298 K temperature). However, there is no water Raman signal observed. This can be explained by the low Raman scattering cross-section of water, so it is below the detection limit in the present experiment. It should be noted that the spectrum shown is a single-shot spectrum to give an impression of the signal-to-noise ratio that can be achieved.

In order to make sure that the observed signals are really Raman and LIBS signatures, several control experiments were carried out as described in the Supplemental Material.

## Conclusion

Combined single-shot Raman/LIBS measurements in the gas phase have been carried out for the very first time. This was enabled by producing a pair of laser pulses with very specific characteristics from a single Q-switched laser. For this purpose, a ring-cavity pulse stretcher was combined with an optical delay line. Three different arrangements were discussed and a proof-of-concept experiment was carried out using a polarization-controlled variant.

A great benefit of the proposed Raman/LIBS technique is that it allows a comprehensive chemical characterization of the sample as it facilitates detection of virtually all molecular and atomic species simultaneously. In the demonstration experiment, the actual measurement took about 2 µs. However, a frank analysis of the temporal sequence shows that the Raman and LIBS signals are generated only about 60 ns after each other, so this is the actual time scale of the measurement.

## Supplemental Material

sj-pdf-1-asp-10.1177_00037028241227459 - Supplemental material for Simultaneous Application of Raman and Laser-Induced Breakdown Spectroscopy in the Gas Phase with a Single Laser and Detector Supplemental material, sj-pdf-1-asp-10.1177_00037028241227459 for Simultaneous Application of Raman and Laser-Induced Breakdown Spectroscopy in the Gas Phase with a Single Laser and Detector by Johannes Kiefer in Applied Spectroscopy
