# Supplementary material for: Simultaneous Application of Raman and Laser-Induced Breakdown Spectroscopy in the Gas Phase with a Single Laser and Detector
Source: Appl Spectrosc. 2024 Jan 31;78(4):438–41. doi: 10.1177/00037028241227459 (PMC10935613; doi:10.1177/00037028241227459)
Supplement: sj-pdf-1-asp-10.1177_00037028241227459 - Supplemental material for Simultaneous Application of Raman and Laser-Induced Breakdown Spectroscopy in the Gas Phase with a Single Laser and Detector [file sj-pdf-1-asp-10.1177_00037028241227459.pdf]

Supplementary Material

for

Simultaneous application of Raman and laser-induced breakdown spectroscopy in the gas phase with a single laser and detector

Johannes Kiefer

University of Bremen, Technische Thermodynamik, Badgasteiner Str. 1, 28359 Bremen, Germany

University of Bremen, MAPEX Center for Material and Processes, 28359 Bremen, Germany

Email: jkiefer@uni-bremen.de

Content:

1) Experimental Setup and Details

2) Control Experiments

1) Experimental Setup and Details

Fig. S1 shows the schematic experimental setup. Pulse stretcher and delay line are represented as a black box, the interiors of which are shown in Fig. 3c of the main manuscript. A frequency-doubled Nd:YAG laser (Quantel, Q-Smart 850, 532 nm) was employed as the light source and an intensified CCD camera (Andor, iStar) was the detector, which was attached to an imaging spectrograph (Princeton Instruments, Acton). The laser was operated at 5 Hz repetition rate and emitted pulses with ~6 ns duration and ~150 mJ energy.

The half-wave plate at the entrance of the pulse stretcher was oriented in such a way that the final Raman pulse had an energy of about 100 mJ (stretched to about 30 ns) and the LIBS pulse had about 25 mJ energy. For completeness, we note that the minimum pulse energy needed for breakdown of the probed gas was 21 mJ.

The beam was focused into the measurement volume with a 200-mm focal length lens (L1). The signal was collected and collimated with an achromatic lens (L2, f = 100 mm), spectrally filtered with a dichroic long-pass edge filter (IF, 550 nm cut-off) and then focused onto the slit of the spectrograph with 250-mm focal length achromatic lens (L3).

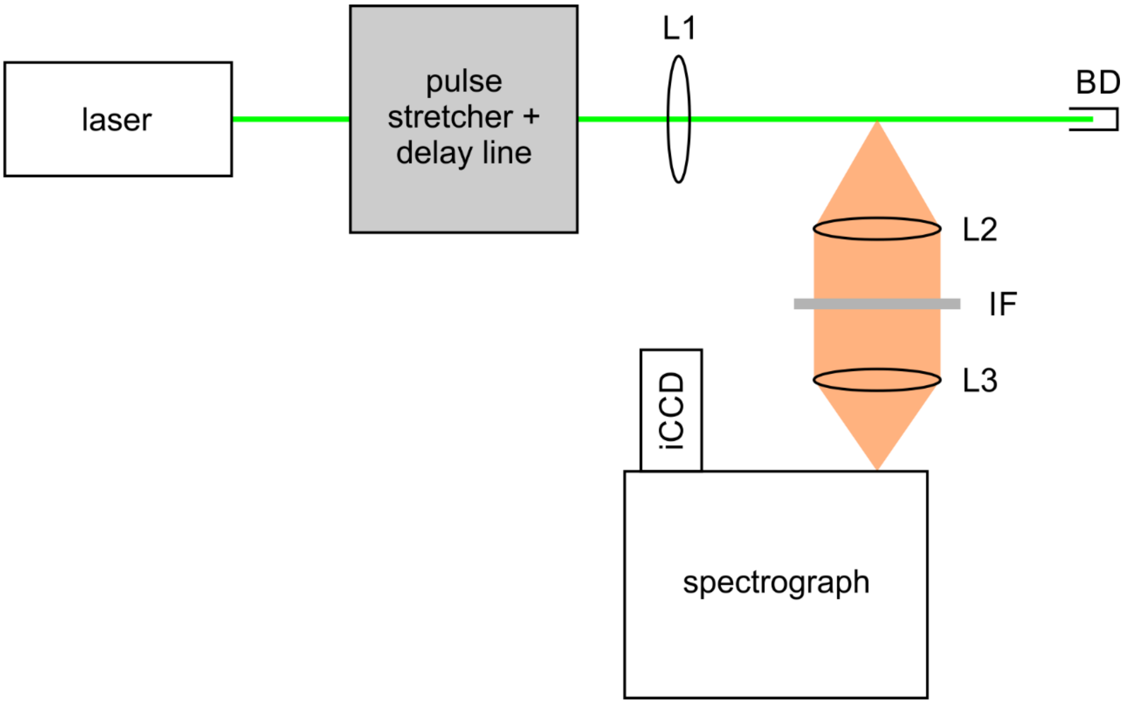

Fig. S1: Schematic experimental setup. L1-L3: lenses, IF: interference filter, BD: beam dump.

2) Control Experiments

In order to make sure that the observed signals are really Raman and LIBS signatures, several control experiments were carried out:

- Using the intensifier gate: When the intensifier was switched on only during the Raman or LIBS window the respective other signal disappeared from the spectrum.
- Blocking parts of the pulse train: When the intensifier was running with two exposures the individual signal components disappeared when the corresponding part of the pulse train was blocked. The positions at which a beam dump was inserted in the delay line are marked P1 and P2 in Fig. S2. The beam dump in position P1 blocked the Raman pulse and in position P2 the LIBS pulse was blocked.

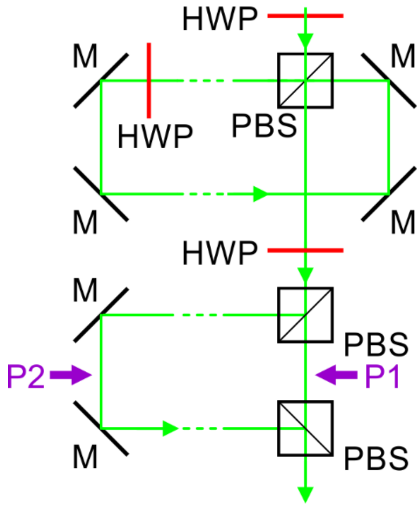

Fig. S2: Schematic PORC pulse stretcher and delay line setup. P1 and P2 indicate the positions where a beam dump was inserted.
